# Gallbladder torsion treated with laparoscopic surgery on the fifth day after onset

**DOI:** 10.1093/jscr/rjaf620

**Published:** 2025-08-12

**Authors:** Toru Takematsu, Asuka Ikeda, Ryota Fukunaga, Keiji Kishikawa, Ichiro Imamura

**Affiliations:** Department of Gastroenterological Surgery, Imamura Hospital, 1523-6 Todoroki-cho, Saga 841-0061, Japan; Department of Gastroenterological Surgery, Imamura Hospital, 1523-6 Todoroki-cho, Saga 841-0061, Japan; Department of Gastroenterological Surgery, Imamura Hospital, 1523-6 Todoroki-cho, Saga 841-0061, Japan; Department of Gastroenterological Surgery, Imamura Hospital, 1523-6 Todoroki-cho, Saga 841-0061, Japan; Department of Gastroenterological Surgery, Imamura Hospital, 1523-6 Todoroki-cho, Saga 841-0061, Japan

**Keywords:** gallbladder torsion, laparoscopy, cholecystectomy, cholecystitis

## Abstract

Gallbladder torsion is a rare condition and has very similar symptoms to acute cholecystitis and is often difficult to diagnose preoperatively. We report the case of a patient who was initially diagnosed with cholecystitis and subsequently underwent surgery with a diagnosis of gallbladder torsion on the fifth day after onset. An 83-year-old woman presented with a 2-day history of gradually progressive lower abdominal pain. Computed tomography showed a gallstone and an enlarged gallbladder. We diagnosed the patient with gallstone cholecystitis and admitted her to hospital. As the abdominal pain had not improved by the next day, we performed a laparoscopic cholecystectomy on the fifth day onset. Intraoperative findings confirmed necrosis of the gallbladder and torsion at the gallbladder neck. Gallbladder torsion may be misdiagnosed as acute cholecystitis. When abdominal symptoms are not severe as in our case, semi-emergency surgery may be a viable option.

## Introduction

Gallbladder torsion is a rare disease in which the gallbladder twists along its axis, causing obstruction of blood flow to the gallbladder [1]. Gallbladder torsion requires emergency surgery because the gallbladder may become necrotic, leading to gallbladder perforation and bile peritonitis [1]. However, because the clinical findings of gallbladder torsion are similar to those of acute cholecystitis, the diagnosis can be difficult and the condition may be misdiagnosed as cholecystitis and treated conservatively [2]. Herein, we report a case of gallbladder torsion in which the patient presented with minor abdominal symptoms and underwent semi-emergency surgery on the fifth day after onset.

## Case report

An 83-year-old woman was referred to our hospital with a 2-day history of gradually progressive right lower abdominal pain. On abdominal examination, she had spontaneous pain and mild tenderness in the right lower quadrant. She had a medical history of chronic heart failure and chronic atrial fibrillation.

**Figure 1:**
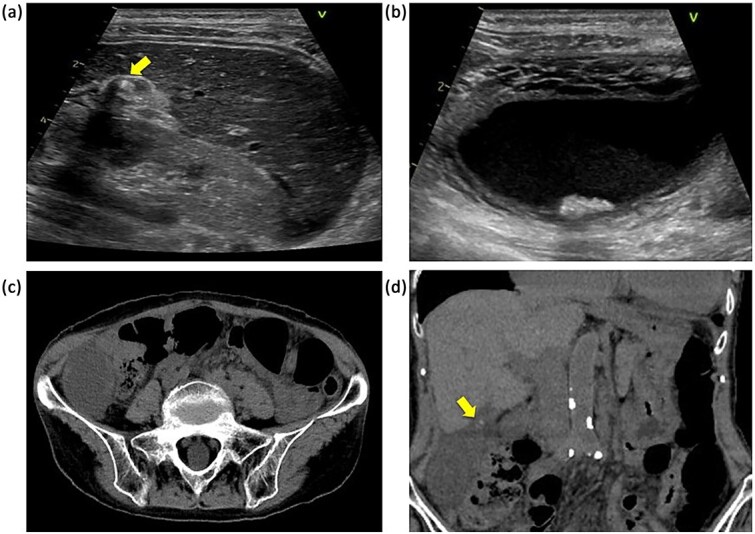
Abdominal ultrasonography and CT findings. (a) Impacted gallstone in the gallbladder neck (arrow). (b) Enlarged gallbladder and edematous thickening of the gallbladder wall. (c) Enlarged gallbladder in the right lower abdominal quadrant. (d) Enlarged gallbladder and gallstone in the neck of the gallbladder (arrow).

**Figure 2:**
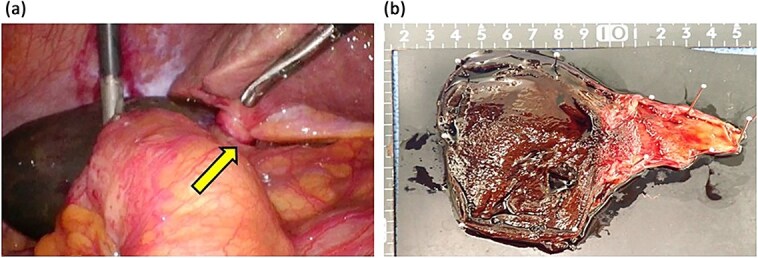
Surgical images and removed gallbladder. (a) Discolored gallbladder and twisted cystic duct (arrow). (b) Surgical specimen.

The laboratory data on admission were: white blood cell count, 7.7 × 10^3^/μl; C-reactive protein concentration, 3.07 mg/dl. Abdominal ultrasound revealed a gallbladder stone in the cystic duct and gallbladder enlargement. The gallstone was located in the neck of the gallbladder (Fig. 1a). The gallbladder wall was thickened in a mesh-like pattern up to 11 mm on the ventral side, and there was lamellar wall thickening of 3 mm on the dorsal side (Fig. 1b). Computed tomography (CT) demonstrated an enlarged gallbladder and a gallstone lodged in the gallbladder neck (Fig. 1c and d). Contrast-enhanced CT was not performed because of the patient’s poor renal function. We initially diagnosed the patient with gallstone cholecystitis, because the abdominal symptoms were not severe and an abdominal ultrasound revealed a gallstone in the cystic duct with thickening of the wall leading to the gallbladder. In addition, given the patient’s history of chronic heart failure and chronic atrial fibrillation, and the fact that she was taking apixaban (Bristol-Myers Squibb Company and Pfizer Inc., Tokyo, Japan), we considered the surgical risk to be high (Charlson comorbidity index score of 6 points) and initiated antibiotic treatment. On the second day after admission, there was no worsening of the inflammatory findings or abdominal symptoms, but the symptoms persisted, so the decision was made to wait until the effects of apixaban had worn off before performing surgery. Preoperative magnetic resonance imaging could not be performed due to scheduling constraints. Laparoscopic cholecystectomy was performed on the third day of hospitalization. Intraoperatively, gallbladder torsion and necrosis were noted: the gallbladder had rotated 180° in a clockwise direction and the cystic duct was obstructed (Fig. 2a). The surgery took 50 min and was completed with minimal bleeding. Due to the high risk to the circulatory system, the patient was managed in a high-care unit after surgery. There were no complications, so she was discharged on postoperative Day 8. Histopathological examination of the gallbladder revealed acute on chronic cholecystitis with transmural neutrophil infiltration, edema, hemorrhage, and necrosis (Fig. 2b).

## Discussion

Torsion of the gallbladder is considered to be most common in elderly women. Gallbladder torsion was first reported by Wendel [3] in 1898 and is said to be caused by rotation of the gallbladder neck and gallbladder duct around its axis under conditions, where the duct is long and the gallbladder is less attached to the liver bed, making it easier for the gallbladder to move [4]. The pathogenesis and clinical symptoms of gallbladder torsion are similar to those of acute cholecystitis, and a preoperative diagnosis of gallbladder torsion is made in only 9.8%–26% of cases [1, 5]. Despite improvements in medical imaging techniques, the preoperative diagnosis of gallbladder torsion remains difficult due to the rarity of the disease and the diversity and non-specificity of its symptoms [6, 7]. The absence of acute onset abdominal pain, poor response to antibiotics, and fever are useful in distinguishing cholecystitis from gallbladder torsion [5]. Our patient was initially diagnosed with acute cholecystitis rather than gallbladder torsion because the onset of the disease was 2 days earlier, the abdominal pain was minimal, and there were gallstones in the neck of the gallbladder. However, the inadequate response to antibiotics led us to suspect gallbladder torsion.

Gallbladder torsion is considered to require prompt surgical intervention [1]. In contrast, cholecystitis not involving torsion can be treated conservatively with antibiotics and percutaneous drainage. However, a delayed diagnosis of gallbladder torsion can lead to gallbladder necrosis and biliary peritonitis due to perforation [8–10]. It has been suggested that cases of gallbladder torsion may undergo surgery on a standby basis, and Kawano *et al.* [7] reported that the mechanism of onset and degree of torsion can be used to divide cases into acute and subacute gallbladder torsion, with subacute cases able to be treated with semi-emergency surgery. The Gross classification system classifies the migrating gallbladder into two types: Type I, in which the gallbladder and ductus are attached to the subhepatic surface by the mesentery, and Type II, in which only the ductus is attached to the subhepatic surface [11]. Type I is incomplete torsion and Type II is complete torsion, with Type II requiring more urgent surgical intervention [7]. In our case, the gallbladder was attached to the subhepatic surface up to the neck of the gallbladder. Our case was Gross Type I, and it was treated as a subacute gallbladder torsion.

Laparoscopic cholecystectomy is the standard treatment for gallbladder torsion and has a good outcome [12]. In the present case, the patient was taking anticoagulant medications. In laparoscopic cholecystectomy, some reports indicate that patients taking antithrombotic drugs do not affect the prognosis, while others report that surgery after drug withdrawal is preferable in patients with a low risk of thrombosis [13]. Therefore, a period of drug withdrawal, if possible, may reduce complications such as postoperative bleeding. Gallbladder torsion is very rare and may be misdiagnosed as acute cholecystitis. As in our case, the abdominal symptoms may not be severe and laboratory data may show little evidence of increased inflammation. In such cases, it may be possible to perform semi-emergency surgery.

## Data Availability

All data generated or analyzed during this study are included in the published article.
